# Clinical characterization of patients with anal fistula during follow-up of anorectal abscess: a large population-based study

**DOI:** 10.1007/s10151-023-02840-z

**Published:** 2023-08-07

**Authors:** E. Sanchez-Haro, E. Vela, M. Cleries, S. Vela, I. Tapiolas, J. Troya, J.-F. Julian, D. Parés

**Affiliations:** 1 Section of Colorectal Surgery, Department of General Surgery, Hospital Germans Trias I Pujol School of Medicine, Universitat Autònoma de Barcelona, C/ Canyet S/N 08916, Badalona, Barcelona, Spain; 2 Àrea de Sistemes d’informació, Servei Català de la Salut (CatSalut), Digitalization for the Sustainability of the Healthcare System (DS3), IDIBELL, Barcelona, Spain

**Keywords:** Anal abscess, Anal fistula, Risk factors

## Abstract

**Purpose:**

Approximately 15–50% of patients with an anorectal abscess will develop an anal fistula, but the true incidence of this entity is currently unknown. The aim of the study was to determine the incidence of anorectal abscess and development of a fistula in a specific population area and to identify potential risk factors associated with demographic, socioeconomic and pre-existing disease (e.g. diabetes and inflammatory bowel disease).

**Methods:**

A longitudinal observational study was designed including a large cohort study in an area with 7,553,650 inhabitants in Spain 1st january 2014 to 31st december 2019. Adults who attended for the first time with an anorectal abscess and had a minimum of 1-year follow-up were included. The diagnosis was made using ICD-10 codes for anorectal abscess and anal fistula.

**Results:**

During the study period, we included 27,821 patients with anorectal abscess. There was a predominance of men (70%) and an overall incidence of 596 per million population. The overall incidence of anal fistula developing from abscesses was 20%, with predominance in men, and a lower incidence in the lowest income level. The cumulative incidence of fistula was higher in men and in younger patients (*p* < 0.0001). On multivariate analysis, patients aged 60–69 years (hazard ratio 2.0) and those with inflammatory bowel disease (hazard ratio 1.8–2.0) had a strong association with fistula development (hazard ratio 2.0).

**Conclusions:**

One in five patients with an anorectal abscess will develop a fistula, with a higher likelihood in men. Fistula formation was strongly associated with inflammatory bowel disease.

## Introduction

Anorectal abscesses are a common surgical emergency treated by incision and drainage [1]. In several series, it has been reported that approximately 15–60% of patients with an anorectal abscess developed a chronic anal fistula during follow-up, which represents a huge variation. Therefore, anorectal abscess had been considered an origin or the cause of a long-term anorectal fistula. In a non-inflammatory bowel disease clinical setting, this clinical process was supported by the cryptoglandular hypothesis [2, 3].

There are limited data on the incidence of anal fistula following anorectal abscess in large populations. In Western countries, the true incidence of this condition has not been calculated and the data provide only an approximation derived from statistical inference based on other European countries [4].

Recently, a well designed study was carried out in the UK using the Hospital Episode Statistics from all National Health Service hospitals in the country. The study demonstrated an incidence rate of 15.5% for anal fistula following an episode of abscess and also indicated the influence of certain factors such as inflammatory bowel disease [5].

Knowledge of the risk factors for developing anal fistula could allow clinicians and health systems to identify those individuals at risk of fistula formation in the future. This information could then be of paramount importance to design strategies for diagnosis and appropriate management of these patients. The information would also be important to update clinical guidelines [6, 7].

Patients with anal fistulas are currently referred to primary care after treatment of the abscess to continue wound healing and follow-up. Therefore, these patients are obliged to seek repeated medical attention before the final diagnosis of anal fistula is made by a proctology specialist. Some risk factors for anal fistula after an episode of anorectal abscess have been described; however, some of them such us diabetes mellitus and socioeconomic status are controversial [6].

The aim of this study was to analyse the incidence of anorectal abscesses in a large cohort of patients in an area of Spain and to identify which patients later developed anal fistula, using a population registry. We also aimed to identify the main risk factors associated with the clinical development of anal fistula after a first episode of anal abscess.

## Materials and methods

### Study design

We performed an observational longitudinal study. Sociodemographic and clinical data were collected from the local health department (CatSalut) dataset of Catalonia (north-east Spain), including all clinical episodes from Catalan hospitals from an area of 7,553,650 inhabitants.

The primary endpoint was the development of an anorectal fistula after an established diagnosis of an anorectal abscess. Therefore, each episode of anorectal abscess was registered and follow-up data were collected from each patient whenever an anal fistula was detected, or at the end of the study period (31 December 2019), or at the time of death (if applicable) January 2022.

### Data source

The Catalan Health Service (CatSalut) provides universal public health coverage to all residents of Catalonia. Since 2011, the Catalan Health Surveillance System has stored diagnostic information collected according to an MDS based on the International Classification of Diseases (ICD-9-CM), which was valid until 2017, when it was replaced by the ICD-10-CM. The registry also includes information on drug prescriptions, outpatient visits, outpatient rehabilitation and many other patient services. It includes an automated data validation system that checks the consistency of the data and identifies potential errors. Moreover, because this information is used for provider payment purposes, external audits are regularly conducted to ensure the quality and reliability of the data. The Catalan Health Service Surveillance system thus provides carefully monitored population-based health data on morbidity and mortality for more than 7.5 million people in southern Europe and has already been used for several research studies [8–10].

### Inclusion and exclusion criteria

All adult patients (aged 18 years and older) with a first clinical episode of anorectal abscess (ICD-9-CM code 566) during the study period (from 1st Jannuary 2014 to 31st december 2019) were included in the analysis. Table 1 lists the codes used for the study. Patients with a diagnosis of an anorectal abscess prior to the inclusion period or who were also diagnosed with an anal fistula at the time of the abscess were excluded from the study (ICD-9-CM code 565.1).

**Table 1:** ICD-10 coding used to register and search diagnosis

Anorectal abscess		Anal fistula	
ICD-10	Clinical diagnosis	ICD-10	Clinical diagnosis
K 61.0	Perianal abscess	K 60.3	Anal fistula
K 61.1	Perirectal abscess	K 60.3	Rectal fistula
K 61.2	Anorectal abscess	K 60.3	Anorectal fistula
K 61.3	Ischiorectal abscess		
K 61.4	Intersphincteric abscess		

### Study variables

We gathered the demographic characteristics of the patients, including age and gender. The patients’ comorbidity burden at the time of anorectal abscess occurrence, mainly diabetes mellitus and inflammatory bowel disease (IBD; Crohn’s disease and ulcerative colitis), was assessed using the health-risk assessment tool for adjusted morbidity groups (AMG), which takes into account the type of disease (i.e. acute or chronic), the number of patients with anal fistula and the complexity of each condition [11–13].

Patients were classified into four AMG strata based on their morbidity-related risk. Baseline risk (healthy stratum) was assigned to the AMG score range, which comprised 50% of the total population. Cut-off points of 50%, 80% and 95% were used to define the population at low, moderate and high, respectively. In addition to morbidity-related risk, we also noted the occurrence of specific diseases that we considered relevant to describe the health status of our population.

Socioeconomic status was stratified into four categories of pharmaceutical co-payment: very low (recipients of social security beneficiaries), low (annual income below €18,000), moderate (annual income €18,000 to €100,000) and high (annual income above €100,000) [11].

### Statistical analysis

Continuous variables are reported as mean (standard deviation) and/or median (interquartile range [IQR], defined as 25th and 75th percentiles) and categorical variables as number and percentage. Comparisons between categorical variables were made using the chi-square test. Survival analysis was performed using the Kaplan–Meier method.

Subsequently, using the variables with a significant difference between categories (Gehan test), a multivariate model was constructed by calculating Cox proportional hazards with robust standard errors. The threshold for statistical significance was set at a two-sided α-value of 0.05 and all analyses were performed in R version 4.0.3 [12, 13].

### Ethical considerations

Because data were collected anonymously from administrative information in a non-personal way, informed consent was not required. The management of data had been done following the European rules of data protection. This study was reviewed and evaluated by the ethics and research committee of our hospital and was supervised according to the Declaration of Helsinki agreements (1975) [14].

## Results

### Sample characteristics and incidence of anal abscess

**Fig. 1:**
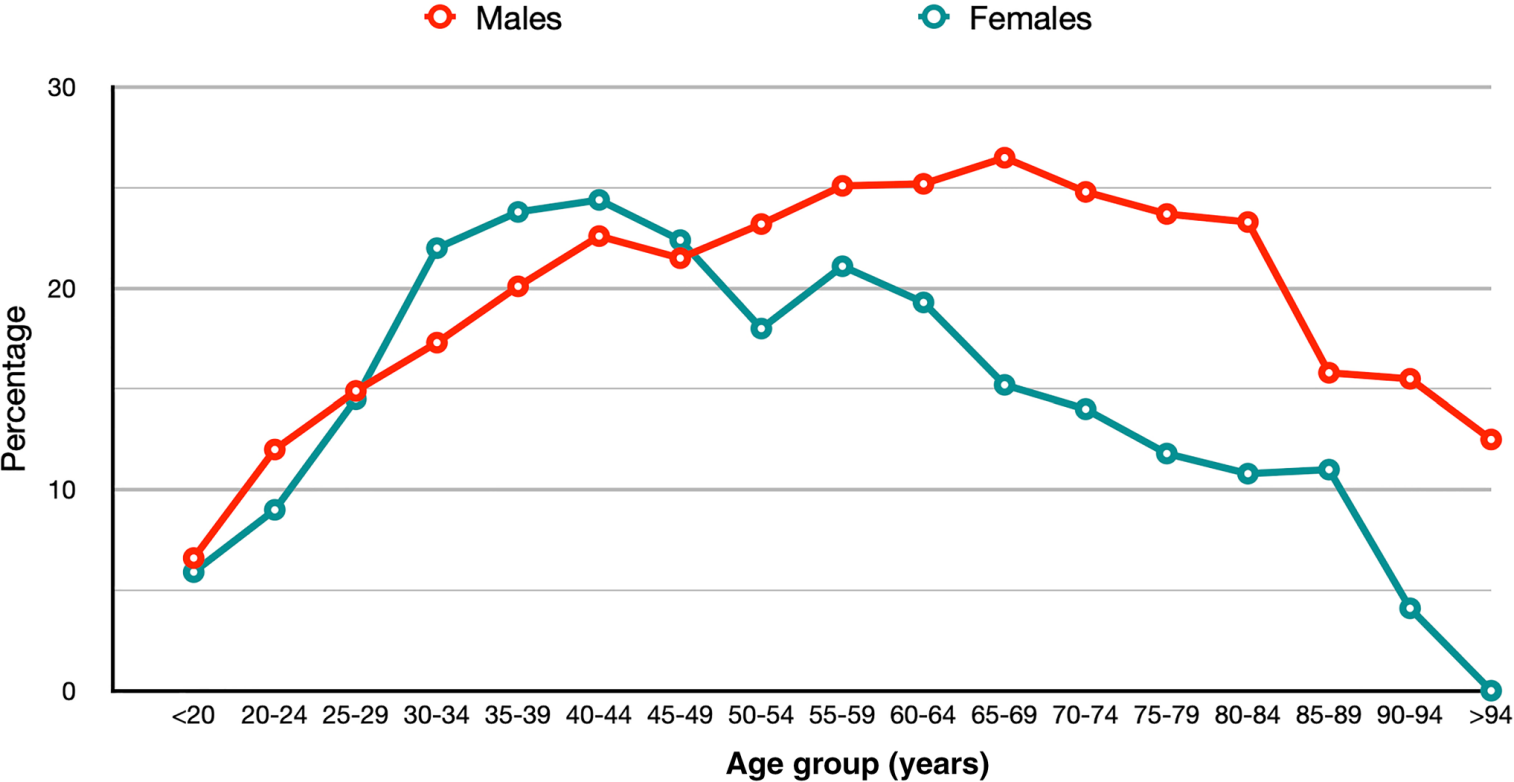
Incidence rate of anorectal abscess according to age group and sex

During the study period, 27,821 adult patients were treated for anorectal abscesses in the population area: 19,437 men (69.8%) (median age 45 years 18-102) and 8384 women (30.2%) (median age 44 years 18-100), who were included in the analysis (Table 2). The overall incidence was 596.5 abscesses per 1,000,000 inhabitants in the year with the highest percentage. During follow-up, 4020 men and 1492 women had an anal fistula identified (Fig. 1).

**Table 2:** Clinical and epidemiological distribution of studied population. HTA: High Blood Pressure; COPD: Crhonic obstructive pulmonary disease; HIV: Human immunodeficiency virus

Characteristics	Population (2014–2019)	Perianal abscess incident cases	Perianal fistula incident cases
Age group (years)			
< 20	851,406	723	45
20–29	4,589,242	4336	563
30–39	6,808,792	5655	1130
40–49	7,533,348	5981	1346
50–59	6,142,222	4818	1101
60–69	4,798,025	3175	749
70–79	3,420,728	1908	395
80–89	2,220,362	1014	166
> 90	561,080	211	17
Total	36,925,205	27,821	5512
Gender			
Male	17,952,845	19,437	4020
Female	18,972,360	8384	1492
Total	36,925,205	27,821	5512
Income level			
Very low	1,921,189	1448	259
Low	28,974,437	19,042	3641
Moderate	14,495,394	7239	1595
High	440,191	92	17
Total	36,925,205	27,821	5512
Main pathologies identified			
HTA	7,606,592	7233	1587
Depressive disorders	4,135,623	4006	728
Diabetes mellitus	2,880,166	3422	623
Asthma	2,437,064	1920	408
COPD	1,698,559	2226	557
Stroke	1,144,681	1057	187
Cirrhosis	184,626	278	61
HIV	147,701	723	154
Ulcerative colitis	110,776	389	138
Crohn's disease	73,850	362	138

The most common comorbidity present in the patient’s cohort was hypertension, followed by a history of cancer and diabetes mellitus (up to 30%). The incidence of anorectal abscess varied by age and sex, with older adults and women being less frequently affected.

The incidence of anorectal abscess declined with age in both sexes. However, in men there were three periods of high incidence with values of 1301, 1183 and 699 abscesses per 1,000,000 inhabitants in the age groups of 25–29, 50–54 and 85–89 years, respectively. In contrast, in women, there were four periods of high incidence in the age groups of 20–24, 40–44, 70–74 and 90–94 years, with values of 677, 513, 337 and 337 abscesses per 1,000,000 inhabitants, respectively.

**Fig. 2:**
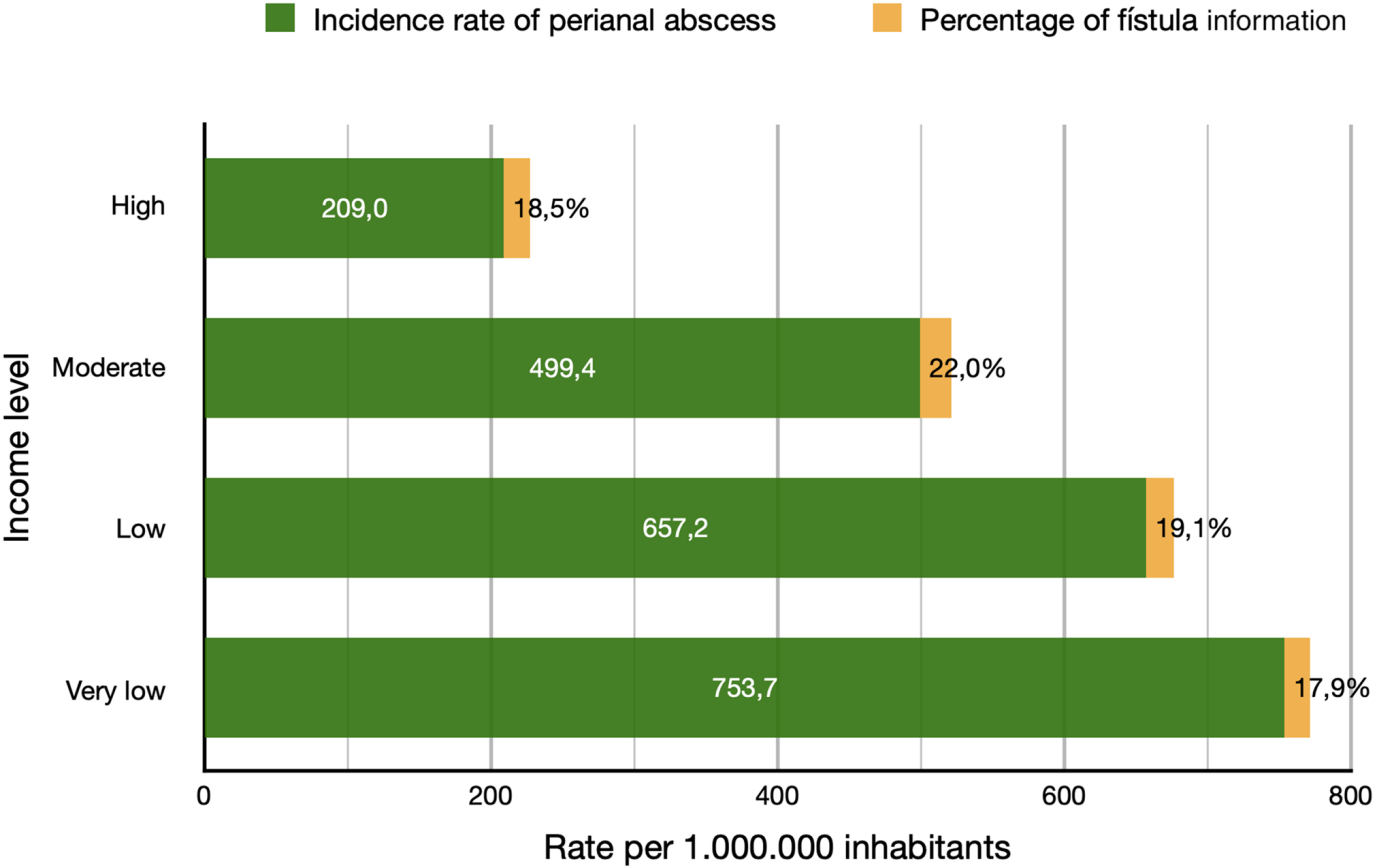
Incidence of anorectal abscess and anal fistula according to income level

The incidence of anorectal abscess also varied by socioeconomic level in Catalonia. The lower the income level was, the higher the incidence rate was. The incidence rate ranged from 209 abscesses per 1,000,000 inhabitants within the high income level to 753 abscesses per 1,000,000 inhabitants in the very low income level (Fig. 2).

### Incidence of anal fistulas after anorectal abscesses

During follow-up, 4020 men (median age 49 years 18-97) and 1492 women (median age 44 years 18-94) were diagnosed with anal fistula, representing 19.81% of patients in our population who developed anal fistula after an initial episode of anorectal abscess.

Figure 1 shows the distribution of the percentages of clinical detection of anal fistula by age group in men and women. The rate was significantly higher in men than in women.

Fistula formation after an abscess also varied by socioeconomic level. Fistulas developed in 22% of patients with a moderate income and in 17.9% of those with a very low income (Fig. 2).

### Timing of anal fistula development

**Fig. 3:**
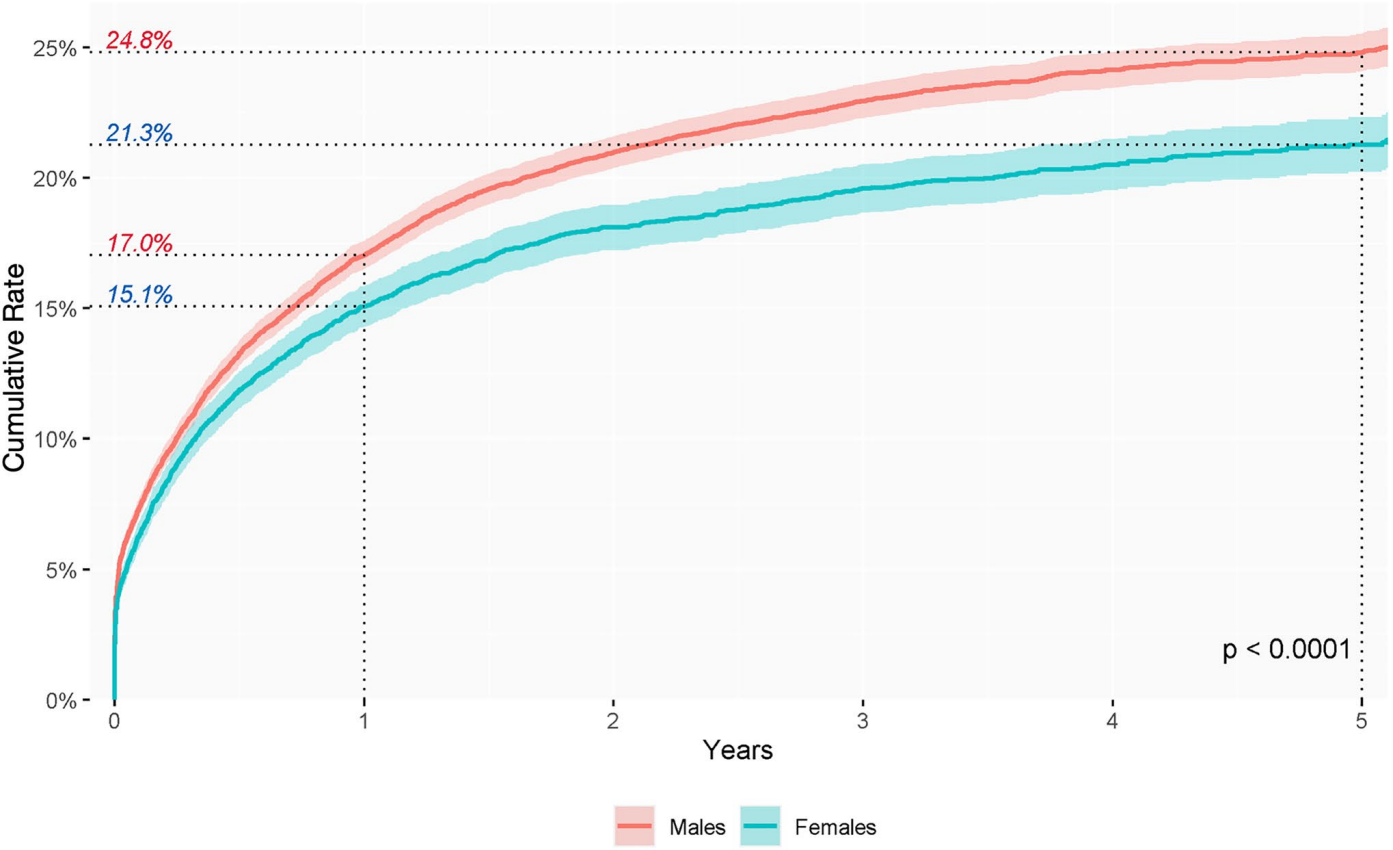
Cumulative incidence rate of anal fistula diagnosis during study period according to sex

The cumulative incidence rate of clinical detection of anal fistula following an episode of anorectal abscess increased in both men and women during follow up. The distribution showed an increasing pattern from the first year of follow-up after the abscess episode. The cumulative incidence rate was 21.3% and 24.8% in the fifth year, for both women and men, respectively (*p* < 0.0001) (Fig. 3).

The cumulative incidence rate of fistula varied by age and was very low in people younger than 30 years compared with other age groups, reaching 9.6% in the first year and 14.8% in the fifth (*p* < 0.0001).

**Fig. 4:**
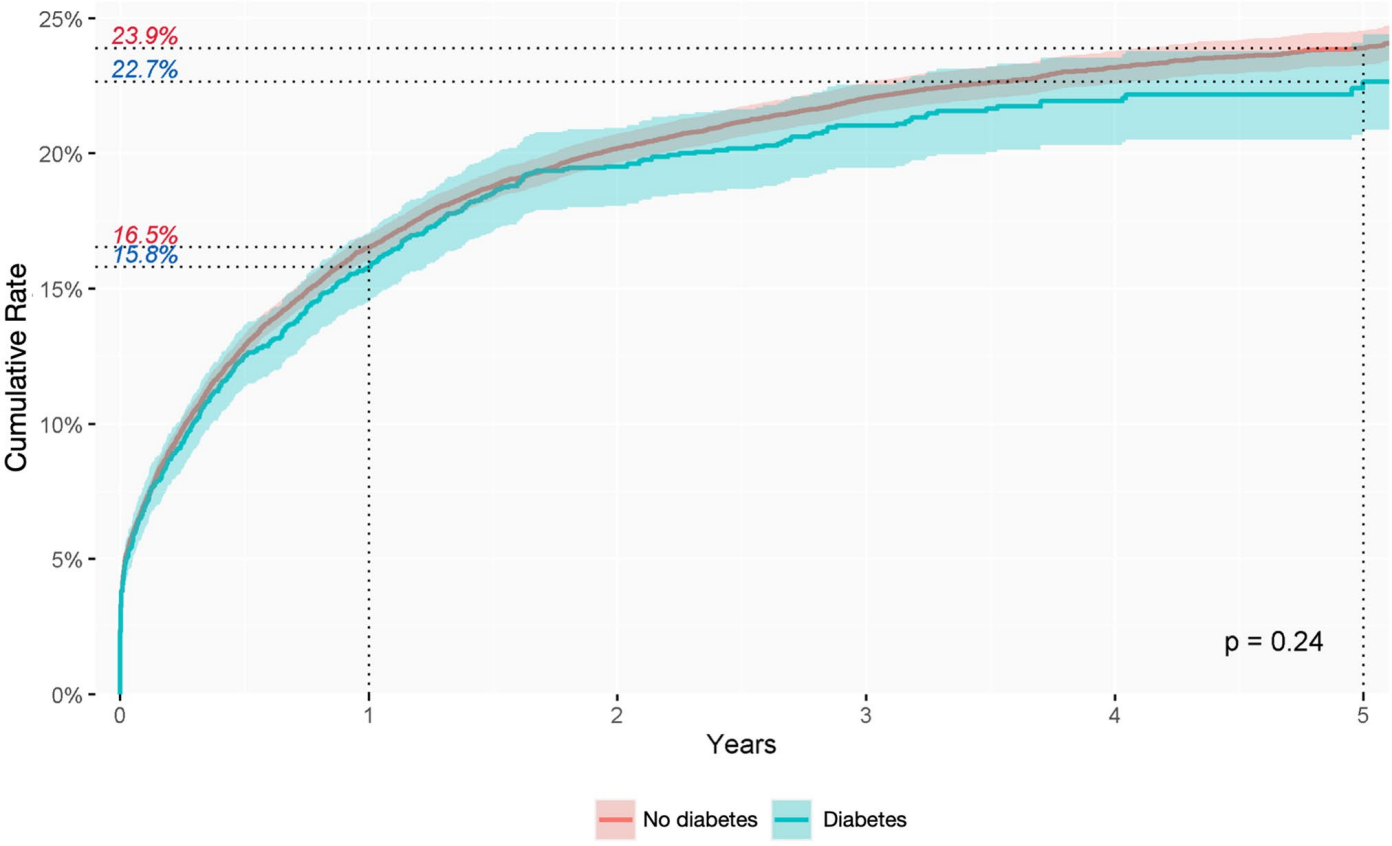
Cumulative incidence rate of anal fistula diagnosis during follow-up in patients with or without diabetes mellitus

**Fig. 5:**
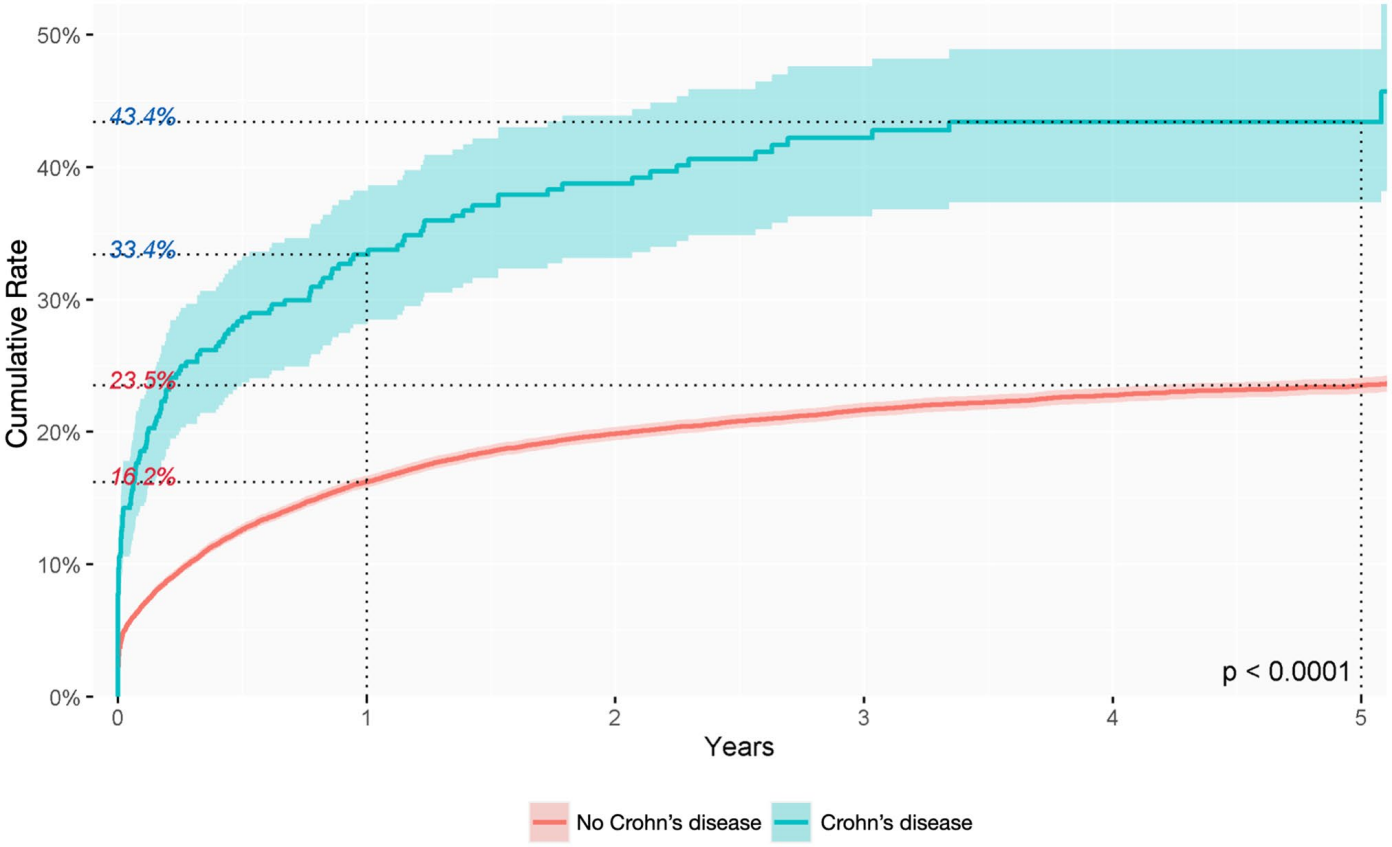
Cumulative incidence rate of anal fistula diagnosis during follow-up in patients with diagnosis of Crohn’s disease

**Fig. 6:**
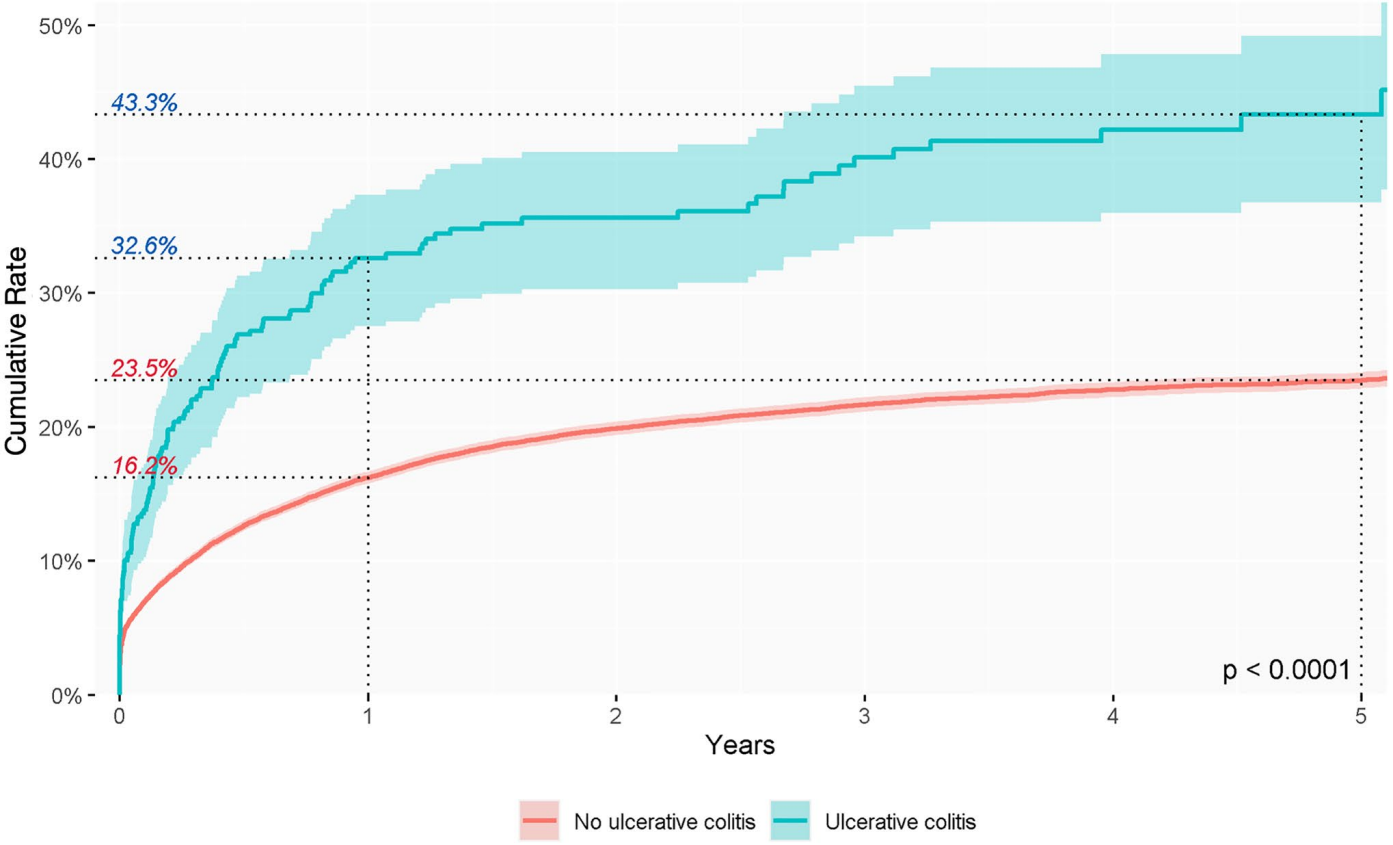
Cumulative incidence rate of anal fistula diagnosis during follow-up of patients with a diagnosis of ulcerative colitis

In this study, the clinical conditions possibly associated with the development of anal fistula were diabetes mellitus and inflammatory bowel disease (IBD). The cumulative incidence was lower, but not significantly so, in patients with diabetes mellitus than in those without (*p* = 0.24) (Fig. 4), while the cumulative incidence rate was significantly higher in patients with IBD than in the general population (Figs. 5 and 6). These features differed slightly depending on the type of IBD. The cumulative incidence rate varied little between distinct types of IBD, being 33.4% in the first year and 43.4% in the fifth year of follow-up in patients with Crohn’s disease (*p* < 0.0001) and 32.6% in the first year and 43.3% in the fifth in patients with ulcerative colitis (*p* < 0.0001).

### Risk factors for anal fistula

**Fig. 7:**
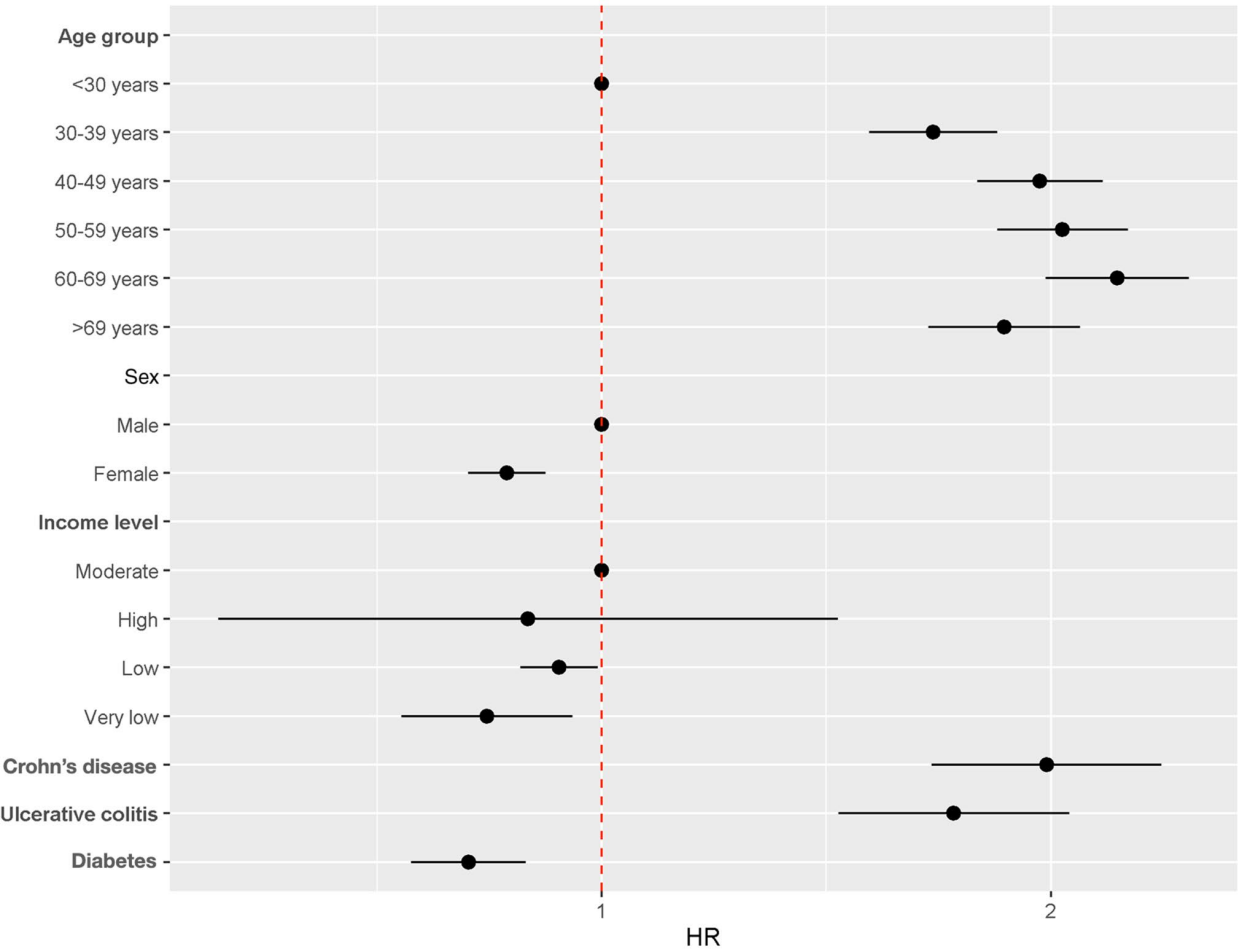
Risk factors for fistula development after anorectal abscess

The results of the multivariate analysis are shown in Fig. 7*.* Age was strongly associated with the development of fistula among patients aged 60–69 years (HR 2.05, 95% CI 1.82–2.30). Other age groups with a significantly higher incidence of fistula development were those aged 40–49 years (HR 1.96, 95% CI 1.78–2.16) and those aged 50–59 years (HR 1.96, 95% CI 1.77–2.18). In addition, sex also affected the risk of abscess formation, with women having a lower likelihood of developing a fistula after abscess formation (HR 0.88, 95% CI 0.83–0.93).

The risk of fistula development after anal abscess was significantly lower in persons with very low incomes (HR 0.86, 95% CI 0.75–0.98) and in those with diabetes mellitus (HR 0.79, 95% CI 0.72–0.87).

In contrast, IBD increased the risk of developing fistula, which was significant in patients with ulcerative colitis (HR 1.76, 95% CI 1.47–2.11) and even higher in those with Crohn’s disease (HR 1.98, 95% CI 1.66–2.37).

## Discussion

From 2014 to 2019, there were 27,821 new cases of anorectal abscesses in Catalonia (mean incidence of 618.2 per 1,000,000 inhabitants per year) and 5512 anal fistulas during follow-up. Thus, approximately one in five patients (19.8%) with abscess developed a clinically detectable anal fistula. In addition, risk factors for the development of an anal fistula were age, gender, socioeconomic status, some comorbidities and prior diagnosis of IBD.

A similar study to our own, based on a national database in England with a 15-year follow-up, calculated an incidence of 202 abscesses per 1,000,000 inhabitants and a progression rate from abscess to fistula of 17.2% [5]. Another study of anorectal abscess recurrence in a cohort of patients who completed 12 years of follow-up and based on data from the Swedish National Patient Registry reported an incidence of 161 per 1,000,000 inhabitants per year [15]. These figures indicate differences between countries, which could be attributed to several factors such as genetics, diet and some as yet unknown factors.

There is wide variation in the incidence of fistula among countries. The first published series calculated a mean incidence of anal fistula of 86 per 1,000,000 inhabitants, 104 per 1,000,000 inhabitants and 232 per 1,000,000 inhabitants in Finland, Spain and Italy, respectively [16]. A systematic review and population-based database analysis to estimate the incidence and prevalence of anal fistula by aetiology (Crohn’s disease, cryptoglandular, traumatic or other) in European countries reported a global incidence rate estimate of 115 per 1,000,000 population per year [4].

Interestingly, we also observed a wide range of percentages of fistula formation after anal abscess in the Catalan population during the study years. This variation has also been reported in other series. A possible explanation is given by the hypothesis of climate changes, but this could be related to mistakes in codification or misdiagnosis due to the difficulty of identifying a fistula. We therefore believe that these results should be interpreted with caution.

Anorectal abscesses and fistula formation in the population in Catalonia have been clinically defined. In the present study, we found higher rates of anal fistulas in men (69.9%) than in women (30.1%). In addition, this clinical condition was especially present in the fourth decade of life in patients with associated comorbidities (a past medical history of hypertension, cancer and diabetes mellitus).

Patient age was identified as a strong factor in the diagnosis of anal fistula, but with different cut-off values. We found a significant association between patient age and fistula formation among patients older than 30 years (with the highest incidence in patients aged 60–69 years), but a similar study with data from 158,713 individuals found the highest incidence in patients aged 41–60 years [5]. Another study, with a series of 148 patients identified age as a risk factor for fistula formation (age group < 40 years, *p* < 0.01) on univariate and multivariate analyses [6]. These latter findings have been supported by recent studies [17].

Previous studies have hypothesized the existence of a hormonal effect on abscess/fistula formation supporting the observation of a higher incidence of this clinical condition in men [5]. We found that incidence of anorectal abscesses was more likely in men (19,437 (69.8%) abscesses) than in women (8384 (30.2%) abscesses). In addition, anal fistula formation was more frequent in men (4020 (20.7%) fistulas) than in women (1492 (17.8%) fistulas). These data, as well as those from other studies, were supported by the results of our univariate (*p* < 0.0001) and multivariate analyses of risk of fistula formation in women (HR 0.88, 95% CI 0.83–0.93). The data are also supported by the results of a previous study [5]. That study hypothesized the existence of gender-based differences, based on a previous study that considered anatomical differences among men and women [5, 18]. Other hypotheses have been postulated, such as possible differences in the types of abscess affecting men vs. women [7] and the aforementioned role of hormones in the local perineal area. Along these lines, a previous study analysed samples of anal anatomy from men and pre- and postmenopausal women. The authors reported a higher presence of all oestrogen, progesterone and androgen receptors in smooth muscle and epithelial tissue in women than in men. Although the sample size was very small, these data suggest a possible role of feminizing hormones in the development of different anal problems [19]. Interestingly, other studies have found that women with anal fistulas had significantly higher circulating oestradiol levels (*p* = 0.03) and lower progesterone levels (*p* = 0.039) than female controls [19, 20]. However, these studies did not find a positive association between oestrogen/oestradiol action and fistula development.

Socioeconomic status is a controversial issue that has been associated with several clinical disorders. An explanation of how it may influence different conditions is a matter of debate. Our multivariate analysis identified a significantly lower association between very low income and fistula development. A previous study found a linear correlation between fistula incidence and income, with more frequent anal fistula development in Q1 (high socioeconomic status) and less frequent fistula formation in Q5 (very low socioeconomic status) [5]. There are several hypotheses that explain this result. First, the low-income population may have less access to health services. However, other factors such as diet and faecal microbiota have also been suggested to play a role in fistula pathogenesis [5, 6].

It is well known that there is a strong association between IBD and the risk of anal fistula development. Although many studies have reported the importance of fistulous anorectal disease in IBD, there is limited epidemiological information on the progression of abscess to anal fistula in patients with IBD in the general population. Classically, anorectal disease has been reported in 5% of patients with IBD [5, 21]. Specifically, anal fistula was diagnosed in 47% of patients with Crohn’s disease and in 26.7% of those with ulcerative diagnosis [22]. Our results show that the percentage of fistula formation during the first year of follow-up after an anal abscess is similar in patients with Crohn’s disease and in those with ulcerative colitis (33.4% and 32.6%, respectively). Our multivariate analysis showed that patients with IBD had an almost twofold risk of fistula development (HR 1.98 and 1.76 for Crohn’s disease and ulcerative colitis, respectively). These findings are supported by those of another study confirming that abscesses were more likely to lead to fistula in patients with IBD, with the median time to fistula diagnosis decreasing from 7 to 5 months, especially in Crohn’s disease [5]. Other studies have found no association between a diagnosis of IBD and risk of anal fistula [17]. The similar percentages of anal fistula in both types of IBD in our study should be analysed with caution. The cause of anal fistula in these patients may be multifactorial, including genetic, immunological and microbiological factors and is likely the result of a deep penetrating ulcer in the anorectum [5, 22]. Therefore, our data could represent a codification misdiagnosis or, as previously suggested, the effect of improved treatment of patients with ulcerative colitis, which may have increased the diagnosis of cryptoglandular-type fistulas in these patients [5].

The influence of diabetes mellitus on anal fistula formation after anorectal abscess has been investigated for the last few decades but it is still a matter of debate. There is information on worse outcomes of anal fistula surgery in these patients, but little or no information on the influence of diabetes on fistula development. In our study, diabetes was an important comorbidity in incident cases of abscess and fistula. However, multivariate analysis showed that diabetes mellitus as an isolated risk factor was associated with a lower risk of fistula development (HR 0.79, 95% CI 0.72–0.87). In another series, patients without diabetes had a two- to sevenfold higher risk of recurrence of anorectal sepsis than patients with diabetes in a univariate analysis (*p* = 0.04) [6]. However, in a smaller study, diabetes mellitus was associated with a lower likelihood of developing fistula in a multivariate analysis (OR 0.5, CI 0.3–0.9; *p* = 0.027) [7]. A more recent study concluded that all patients with diabetes have an increased risk for anorectal abscess but not for recurrent disease [15]. Finally, a recent study of a large population-based Swedish registry found that patients with type I diabetes had a lower rate of anorectal abscess than those with type II diabetes after adjustment in a multivariate analysis (OR 0.65, CI 0.6–0.7) [23]. In addition, the authors of that study found that poor glycaemic control was directly proportional to abscess formation, suggesting that metabolic breakdown may be more important than autoimmune factors in its pathogenesis [23]. Thus, prospective trials are warranted to investigate the relationship between diabetes and anorectal abscess–fistula formation.

This study is a general population study with a large sample that provides information on the real incidence of anal abscesses and the correlated incidence of anal fistulas in some risk groups, and adds to the data provided by previous studies with similar methodology [5]. However, there are some limitations. First, the analysis was retrospective and diagnosis of anorectal abscess and anal fistula formation was drawn from clinical registries of the public funded health system. The latter is relevant to the value of information related to influence of socioeconomic status. It would be interesting to have information on the type and other clinical details of each episode of abscess but, as in other published series, this clinical information was not available. Finally and despite the lack of details for each study variable, we identified some risk factors, which may help scientific societies to design a specific follow-up for these patients.

## Conclusion

Anal fistulas are common during clinical follow up of an anorectal abscess. In this study, fistulas developed in almost one in five patients with anorectal abscess. The incidence of anal abscess and fistula was significantly higher in men than in women and in specific age groups. Socioeconomic status did not play a clear role in fistula formation and diabetes may be related to lower rates of fistulation. Finally, the incidence of anal fistula was strongly influenced by the presence of IBD during follow-up.

## Data Availability

The data that support the findings of this study are not openly available due to reasons of sensitivity and are available from the corresponding author upon reasonable request. Data are located in controlled access data storage at Hospital Germans Trias i Pujol (https://www.hospitalgermanstrias.cat/servei-cirugia-general-digestiva?info=iconPresentacion).
